# Systemic Intervention Through Relational Savoring: Cultural Considerations for Working With Asian American Families

**DOI:** 10.1111/jmft.70080

**Published:** 2025-10-05

**Authors:** Frances Haofei Li, Elayne Zhou, Hans Oh, Jessica L. Borelli

**Affiliations:** ^1^ Department of Psychology University of California, Irvine Irvine California USA; ^2^ Department of Psychology University of Southern California Los Angeles California USA; ^3^ Suzanne Dworak‐Peck School of Social Work, University of Southern California Los Angeles California USA

**Keywords:** Asian American, caregivers, cultural adaptation, relational savoring, systemic therapy

## Abstract

Relational savoring (RS) is a brief, accessible, relationship‐based intervention guided by systemic principles. RS may benefit Asian American, Native Hawaiian, and Pacific Islander (AANHPI) families experiencing relational difficulties. Family ties are often protective for AANHPI groups, yet acculturation‐related stress may impede families' ability to leverage this asset. RS has evidenced effectiveness in increasing well‐being and relationship quality, but has yet to be tested with AANHPI families. RS may align well with cultural values relevant to AANHPI communities, such as interconnectedness, while also overcoming barriers through its strengths‐based approach. This paper outlines the decision‐making process used to adapt RS for AANHPI families and emphasizes the role of community feedback. An iterative and rigorous approach is essential to determine whether adaptation is needed and to identify appropriate targets for change. Importantly, enhancing the cultural responsiveness of RS furthers our goal of promoting flourishing and addressing health disparities among underserved families.

## 1 Introduction

The Asian American, Native Hawaiian, or Pacific Islander (AANHPI)^1^ population is rapidly growing, yet there is evidence that many are languishing more than other ethno‐racial groups (Oh 2023). This is concerning in light of findings that suggest AANHPI individuals tend to underutilize formal support services, highlighting a critical treatment gap (Abe‐Kim et al. 2007). Addressing these treatment disparities necessitates thoughtful and culturally responsive interventions. A systemic approach may uniquely position practitioners and researchers to respond to the diverse and complex needs of this population.

In this paper, we underscore the importance of a systemic approach when treating AANHPI clients, with a focus on AANHPI caregivers. We then highlight a promising emerging intervention, *relational savoring* (RS; Borelli 2024), describing its mechanisms and theoretical foundations. We then explore considerations behind cultural adaptation, weighing arguments for and against adaptation, as well as the processes recommended for evaluating and pursuing adaptation within the AANHPI community. In addition to their applicability to RS, the ideas discussed here can be used to guide thinking regarding the adaptation of systemic and other strengths‐based interventions for AANHPI groups.

### 1.1 Systemic Family Therapy (SFT) Approaches

As an approach that conceptualizes the dynamics between individuals as part of an interconnected interpersonal system (Dallos and Draper 2015), the SFT approach may have particular relevance for communities that prioritize cultural values around interdependence (i.e., construing self as interdependent to others and emphasizing interpersonal harmony; Markus and Kitayama 1991), such as many AANHPI communities. These cultural values lend themselves to a stronger identification with a broader ingroup (e.g., family, racial/ethnic group) and a less clear distinction between the self and the group. SFT practices may be used to tap into the existing group‐level strengths of AANHPI clients by orienting individuals to their positive connectedness within the family system.

SFTs have been applied with AANHPIs such as South Asian American immigrants (Rastogi 2007) and with Asian families such as Chinese Singaporeans (Sim et al. 2017). However, to our knowledge, the effectiveness of SFTs with AANHPI families has only been reported in case studies. For example, Rastogi (2007) administered emotionally focused family therapy (Johnson and Lee 2005) to two Indian American families with consideration of their interconnectedness and commitment to intergenerational relationships. Following the intervention, both families evidenced conflict resolution and relationship improvement, while honoring and maintaining their values. While we do not yet have empirical evidence of efficacy, the fit between cultural values and SFTs suggests a promising approach for AANHPI families.

### 1.2 RS and Connections to Systemic Approaches

RS (Borelli 2024) is a brief, adaptable, and accessible systemic intervention in which interveners assist clients in reflecting on and re‐experiencing a moment of positive connectedness with another person. RS is considered to be a systemic intervention in that it targets relational, family‐level outcomes (closeness, relationship satisfaction, interpersonal sensitivity) as its primary therapeutic targets by helping people focus on moments of close interpersonal connections. RS is purported to achieve these outcomes via four mechanisms of change (Borelli, Smiley et al. 2020): (1) cultivate positive emotions and derive meaning from relationships; (2) promote mentalization (i.e., the capacity to understand one's own and others' mental states; Luyten et al. 2020); (3) enhance awareness of sensitive behaviors; and (4) increase vulnerability in relationships. For a figure illustrating RS's mechanisms of change and intended outcomes, please refer to Borelli, Smiley et al. (2020).

Systemic therapies may involve working with multiple family members (e.g., functional family therapy; Sexton and Alexander 2003) or a single individual (e.g., narrative therapy; Cowley and Springen 1995) provided the focus remains on relationships. Similarly, although RS was originally designed for individual delivery, it has been adapted for use with dyads and groups to better align with the needs of different populations. Regardless of delivery modality, RS focuses on the individual's broader relational context, seeking to bolster relationship qualities in the broader relational system. For example, in an iteration of RS designed for caregivers (Borelli 2024), interveners guide clients to focus on a moment in which they provided sensitive care (i.e., care that appropriately and effectively responded to a need; e.g., when the caregiver comforts his son after he is frightened). Furthermore, studies have shown that delivering RS to parents can positively impact child outcomes, indicating that RS interventions can benefit both members of the parent–child dyad (Blackard 2024). In another iteration of RS designed for delivery with Latine‐serving community health workers, interveners guide clients to savor moments of supporting their community (Arcos et al. 2023; Zhou et al. under review).

#### 1.2.1 Accessibility

RS can be implemented by paraprofessionals (individuals without bachelor's degrees) such as community health workers (Borelli, Yates, et al. 2020) or undergraduate research assistants (Borelli et al. 2023) with high levels of fidelity (Borelli, Perzolli, et al. 2024). The training process for RS delivery involves 4 h of didactics, recorded practice, review and feedback, certification, and weekly consultation meetings. RS accommodates a variety of accessible delivery methods, including home visits (Borelli et al. 2023), internet administration (Burkhart et al. 2015), group therapy sessions (Ansarifar et al. 2025), and in an app‐based format (Nguyen et al. 2025). Research trials on self‐administered RS reveal promise in terms of their impact in the short‐term (Borelli et al. 2014; Burkart et al. 2015), as well as their acceptability, and have a high potential for dissemination (Nguyen et al. 2025).

#### 1.2.2 Stages of Intervention

The RS intervention consists of three stages: mindfulness exercise, memory selection, and memory reflection (Borelli 2024). RS begins with a brief mindfulness exercise (paced breathing) to facilitate a calm state and prepare the client to engage in the reflective process. Next, the intervener assists the client in recalling relationship memories that are rich in attachment content or positive connectedness and identifies the most appropriate one for savoring. Together with the client, the intervener selects a specific memory that contains (1) attachment content, including when the care‐taker is acting as a secure base (from which the care‐receiver can explore novel environments) or safe haven (to which the care‐receiver can return for reassurance; Bowlby 1983) and (2) positive emotionality and emotional connectedness, to avoid negative emotions “spoiling” the savoring process. If clients select a memory involving intense negative emotions, the intervener guides the client in selecting an alternative memory. In the final memory reflection stage, the intervener elicits reflection from the client across five domains, including sensory reflection, emotion reflection, meaning making, future focus, and open‐ended mind wandering. Similar to the memory selection stage, should the memory reflection process begin to focus on negative aspects of the interaction, the intervener will redirect the client to focus on positive moments of connection.

#### 1.2.3 Theoretical Foundation

RS is premised on theoretical tenets of positive psychology (Seligman et al. 2005), which recognize the central role of positive emotions in adaptation and functioning. Fredrickson's broaden‐and‐build theory holds that positive emotions, which are closely connected with positive relationships, broaden perspectives and foster growth and resilience (Fredrickson 2004). While traditional psychological interventions primarily target symptoms, positive psychology interventions recognize the presence of pathology and seek to bolster positive emotions and experiences (Magyar‐Moe 2009). RS interveners leverage positive relationship memories to promote reflection, growth, and openness to intervention. RS is also guided by attachment theory (Bowlby 1983), which posits that early interactions with attachment figures (i.e., key early caregivers) shape internal working models (i.e., mental representations of the self and attachment figures; IWMs). These IWMs help individuals interpret information from social interactions through the lens of prior experiences. RS aims to facilitate a deliberate focus on positive relationship memories, broadening the information available to the IWM, and fostering an appreciation of the positive aspects of relationships (Borelli 2024).

In addition to its foundation in positive psychology and attachment theory, RS closely aligns with the principles of systemic theory and various family therapies. First and foremost, RS focuses on moments of connectedness within relationships. Second, in accordance with the principles of systemic theory, RS reflects the systemic principle that care is reciprocal by aiming to improve individuals' capacity to receive care by enhancing their awareness of providing care (and vice versa). Third, similar to emotionally focused couples therapy (Greenberg and Johnson 1988), RS highlights the importance of attachment needs in relationships and the role of emotions in shaping attachment behaviors. RS guides clients to express vulnerable emotions related to their attachment needs with significant others to fortify their relationships. Consistent with strengths‐based systemic approaches (e.g., narrative therapy; Cowley and Springen 1995), RS also emphasizes tracking positive relational behaviors and reducing the focus on negative ones.

#### 1.2.4 Evidence Base

RS has garnered evidence for its efficacy and effectiveness from over two dozen studies across various settings and populations, with many conducted by independent investigators, meeting criteria to be considered a psychological treatment with a strong evidence base (Chambless and Hollon 1998; Tolin et al. 2015). RS is effective when implemented with individuals across the life span (e.g., Smiley et al. 2024) and in different relationship contexts (parents, adult romantic partners; Burkhart et al. 2015). RS is effective in enhancing positive emotion (Borelli et al. 2023), physiological states (Borelli et al. 2019), relational outcomes (Borelli et al. 2014), adaptive parenting (Ansarifar et al. 2025; Borelli et al. 2023; Smiley et al. 2024), and child emotion reactivity (Blackard 2024). The developers of RS have expressed an interest in evaluating the intervention for its fit with different cultural groups, as well as the desire to adapt to increase cultural congruence (Borelli 2024). To date, RS research has focused on adapting the intervention to be culturally congruent for Latine families by modifying language, including local metaphors, and transitioning to group therapy and telehealth administration based on community members' feedback (e.g., Borelli, Zhou, et al. 2024). Research has not assessed the effectiveness of RS with AANHPI individuals despite its fit with the values of AANHPI communities. Thus, examining the cultural fit with AANHPI individuals is an important goal of RS researchers. Evidence from more independent investigators and meta‐analytic studies will further strengthen the evidence base for RS.

### 1.3 The Need for Culturally Responsive Systemic Family Therapies

While the family system is often the individual's most immediate and influential context, the structure and content of familial relationships are also interwoven with cultural values, beliefs, and practices. That is, according to Urie Bronfenbrenner's (1992) Ecological Systems Theory (EST), individuals (and their identities and attachment styles) exist within and are acted upon by interconnected proximal and distal systems. Further, a recent revision to Bronfenbrenner's theory proposed recognizing culture as a part of the microsystem, emphasizing the inextricability of culture from everyday practices (Vélez‐Agosto et al. 2017).

By only considering the microsystem, and thus only intervening at this level, broader processes that act upon the well‐being of families may be overlooked, limiting the utility of interventions. SFTs, then, must be responsive to individuals' broader cultural contexts and experiences to more effectively support both individual and family well‐being, particularly in racial–ethnic minoritized (REM) groups, lest SF therapists under‐emphasize the importance of cultural influences on well‐being. Simultaneously, we caution SF therapists against over‐emphasizing the role of culture in clients' presentations. We urge therapists to engage in self‐reflection to avoid making harmful assumptions when working with REM clients, such as “culture‐blaming” (i.e., over‐attributing clients' presenting problems to cultural influences) and generalizations that REM clients inherently require different treatment approaches than White clients (i.e., making automatic adaptations based on past experiences with REM clients or assuming that interventions are not acceptable or relevant for REM clients).

Over the past two decades, SF therapists have steadily moved away from notions of cultural competence that inadvertently “classify, categorize, and define ‘others’” (Knudson‐Martin et al. 2020, 619). Clinicians instead practice sociocultural attunement in their delivery of therapies, which Knudson‐Martin et al. (2020) noted includes several characteristics: relational focus, third‐order thinking, responsibility toward equity, and nuanced attention to context. In line with this view, RS maintains a relational focus in eliciting clients' key moments of connection. This allows the client's narratives and contexts to take center stage, empowering clients to engage with and explore those experiences, as well as allowing the therapist to better understand how interpersonal ties are interpreted and experienced by the client.

RS also presents an opportunity for therapists to create space for third‐order thinking, wherein clients are prompted to imagine what futures within their family systems and beyond might be possible as a result of relational decisions made in the present (Knudson‐Martin et al. 2020). RS may promote third‐order change beyond the microsystem. Adapted versions of RS have integrated cultural values of Latine communities, emphasizing social justice and social change, both within and in addition to the RS intervention itself (Arcos et al. 2023; Borelli 2024). RS has been delivered to and by community health workers; these efforts have further built capacity for change in these essential workers, while also shifting expertise to communities. When delivered to community health workers, RS narratives have included discussions of broader legal, justice, and health systems, as well as social models of health (i.e., creating optimal conditions for health beyond medication and symptom treatment, through community caregiving, mutual aid, etc.) (Zhou et al. under review). As such, broader ecological contexts can permeate experiences of RS, particularly in the meaning‐making of impactful interpersonal experiences as well as the future‐focus–that is, an interpersonal interaction may be embedded in larger, systemic dynamics, and future action may also be a collective response (e.g., sociopolitical action, collective care, cultural authenticity, etc.). Finally, RS is accessible, flexible, cost‐effective, open‐ended, and requires a low training burden (Borelli, Perzolli, et al. 2024), allowing therapists to be equity‐ and context‐minded.

Below, we explore the clinical needs of AANHPI families, considerations behind whether or not to adapt RS to meet these needs, and the importance of community‐based participatory research (CBPR) methods in guiding information‐gathering and implementation processes.

## 2 The Clinical Needs of AANHPI Caregivers

### 2.1 Caregiving Strains and Barriers to Care

AANHPI communities are identified as the fastest‐growing ethnic group in the United States, yet the mental health of this population has been overlooked and related research underfunded (Hall and Yee 2012). AANHPI caregivers, those who provide caregiving for children or older adults, face elevated risks for poor health outcomes due to caregiving stress (Pinquart and Sörensen 2005), sociocultural influences (Misra et al. 2021), and systemic barriers (Zane et al. 2005) to accessing support. While caregiving stress is not unique to AANHPI individuals, it may be heightened compared to other groups (National Alliance for Caregiving NAC & AARP. 2020).

#### 2.1.1 Cultural Beliefs in Caregiving

A cultural value commonly identified among AANHPI groups is *familism*, wherein family members are interdependent and the well‐being of the family is considered more important than that of the individual (Y. Choi et al. 2021). In Chinese communities in particular, the cultural value of *filial piety* involves the duty of deference, respect, and care for one's parents and elders. Unsurprisingly, approximately 24% of AANHPI individuals live in multigenerational households compared to 13% of their White counterparts (Cohn et al. 2022), sometimes caring both downwards (i.e., to youth) and upwards (i.e., to older adults) simultaneously (National Alliance for Caregiving NAC & AARP. 2020).

Cultural beliefs regarding family may be a double‐edged sword: while family support is protective against stress (Corona et al. 2017), interdependent attitudes are associated with greater mental health stigma (Zhou et al. 2022), and greater adherence to filial piety is linked to more stress and poorer well‐being (Dong and Xu 2016). Relatedly, family structure and physical proximity encourage more time spent caregiving in AANHPI households, and informal caregiving lends itself to a greater chance of experiencing emotional exhaustion and other dimensions of burnout (Gérain and Zech 2021). Additionally, many AANHPI caregivers report family caregiving as a shared cultural value and role over which they have little control (Pharr et al. 2014). Lack of choice in caregiving is linked with heightened stress and poorer physical health (Schulz et al. 2012). Thus, the cultural embeddedness of caregiving and the importance of family relationships present AANHPI communities with both familial interconnectedness and greater burdens to bear. It is no surprise, then, that AANHPI family caregivers experience depression related to caregiving burden (Liang and Dong 2020). AANHPI caregivers report higher rates of depression than White caregivers (Pinquart and Sörensen 2005), worse relationships with the people they are caring for, and greater emotional stress from caregiving than other groups (National Alliance for Caregiving NAC & AARP. 2020).

#### 2.1.2 Immigration, Acculturation, and Caregiving in the Family System

Many AANHPI caregivers are also tasked with caregiving in the context of immigration, which complicates family dynamics and increases their mental health needs. The majority (71%) of AANHPI adults in the United States are immigrants (Budiman and Ruiz 2021), and face both immigration stress and the ongoing challenges of adjusting to a new culture. This process of acculturation impacts not just the individual but also shakes the entire family system (Portes and Rumbaut 1996). Immigrating and adapting to a new environment is particularly disruptive for youth in key developmental or transitional periods, and is associated with maladjustment (Juang et al. 2012). For example, discrepancies in parenting attitudes and perceptions of youth behaviors (e.g., autonomy as normative or disruptive) can contribute to intrafamilial conflict (Ho 2014). There is also evidence that foreign‐born AANHPI mothers reported greater stress compared to those born in the United States (Nomaguchi and House 2013). Thus, acculturative stress and resultant intrafamilial conflicts strain close relationships and exacerbate psychopathological risks, eclipsing moments of connection for AANHPI families.

#### 2.1.3 Treatment Barriers and Informal Help‐Seeking for AANHPI Caregivers

While AANHPI caregivers may experience significant distress from caregiving, various factors impede their ability to seek and access support. Indeed, AANHPI groups underutilize psychological services compared to the general population (Abe‐Kim et al. 2007). Moreover, those who do seek care often face higher dropout rates than their White counterparts (Green et al. 2020). Multiple structural barriers exacerbate these disparities, such as limited access to health insurance, lack of multilingual services, and a shortage of culturally competent providers (Zane et al. 2005). As such, AANHPI caregivers are more likely to rely on informal support than other racial ethnic groups (Chow et al. 2010). Additionally, AANHPI groups, along with other REM groups in the United States, experience and express greater stigma toward mental illnesses compared to White Americans (Misra et al. 2021). Mental health stigma further deters help‐seeking, causing the under‐utilization of traditional mental health care (Clement et al. 2015). Addressing these multifaceted challenges is imperative in ensuring that AANHPI individuals receive the mental health care they need and deserve. These disparities underscore the pressing need for culturally acceptable and accessible interventions to address the mental health challenges faced by AANHPI communities.

### 2.2 Relationships as a Promising Port of Entry for AANHPI Caregivers

Given the strong cultural emphasis on family ties for many AANHPI caregivers, relationships are a promising target for intervention. Delivering the interventions directly to AANHPI caregivers is less resource‐intensive than SFTs involving multiple members of the family, yet can have cascading effects on the whole system. RS may be particularly appropriate for AANHPI caregivers because it addresses several key barriers (e.g., stigma, cultural mismatch, and accessibility) that have historically impeded AANHPI help‐seeking and treatment utilization. That is, RS has the potential to be non‐stigmatizing due to its general focus on relational strengths and positive connections (Borelli 2024), culturally congruent due to its ability to evoke and reinforce cultural values that emphasize the significance of family connections, and accessible due to its flexibility of delivery (i.e., who delivers it and how). However, no work has explicitly explored the fit of this intervention with the largely heterogeneous AANHPI population. In the following section, we discuss relevant theoretical and practical considerations that may guide RS adaptation considerations for this population.

## 3 To Adapt or Not to Adapt, This Is the Question

To fully leverage RS to serve AANHPI families, one important consideration is whether adaptations of RS for AANHPI clients may be necessary. On the one hand, cultural differences exist across various orientations between AANHPI cultures and other cultures (e.g., individualism vs. collectivism, Schimmack et al. 2005). On the other hand, we should be careful not to over‐attribute individual differences to culture, as it may engender differential treatment of individuals in different cultural groups (Causadias et al. 2018). Further, a formal adaptation to the RS manual may not address the great variability that exists within AANHPI groups themselves. For example, although much psychological and epidemiological research groups people from Vietnam and Laos into the same overarching category of Southeast Asia, their cultural values, financial earnings, and health outcomes are different, underscoring the heterogeneity within this group (Kochhar and Cilluffo 2018).

Several frameworks exist that may guide the cultural adaptation of evidence‐based treatments (EBTs). For one, Lau (2006) proposed the selective and directed cultural adaptation framework to understand the generalizability of EBTs and determine whether adaptations are warranted for diverse populations. *Selective* suggests that cultural adaptations of treatments should be conducted judiciously. Adaptations may be warranted if empirical evidence suggests between‐group differences in (a) contextual risk and resilience factors or (b) response to treatment. *Directed* suggests using data to inform specific targets of adaptations. Both components of Lau's approach highlight the importance of using empirical evidence to guide adaptation decisions and processes. Other research by Hall et al. (2019) identified several types of cultural adaptations made by individual clinicians for Asian and AANHPI clients, cultural adaptations to enhance alliance building and therapist credibility, such as gift‐giving (e.g., conceptualizing therapy to focus on a culturally salient goal as a “gift”); interdependent construal of the self (e.g., therapy focusing on family systems and social contexts); and an awareness of indirect or nonverbal forms of cultural communication. While highlighting the need for future research, we raise several considerations for the potential adaptation of RS for AANHPI families.

### 3.1 Potential Reasons Against Adaptations

#### 3.1.1 Congruence between RS and AANHPI Values

One reason not to adapt an intervention would be if it is already consistent with the values of the targeted cultural group. As noted, RS reflects several of the central values in AANHPI communities (Smith et al. 2019), by tapping into the existence of individuals in relational contexts, the provision and receipt of care, the emphasis on positive affect related to interpersonal relationships, and the strengths‐based, non‐stigmatizing approach. RS may be congruent with filial piety such that clients would be more willing to engage in positive reframing of caregiving as an activity that honors the parents. In contrast, clients may be more reluctant to discuss negative aspects related to caregiving with the intervener out of concern for disrespecting the parents. It may be the case that filial piety ultimately reflects a deep connection to one's parents that, under certain circumstances, can serve as a strong basis for RS. Last but not least, RS may also bridge the accessibility issue commonly faced by AANHPI individuals with its flexibility across delivery modes (in‐person, home visit, over the internet, in an app).

#### 3.1.2 Adaptations May Not Provide Incremental Validity

Another often underappreciated consideration is that cultural adaptations may produce similar or even iatrogenic effects compared to the original intervention. Rigorous testing is needed to determine whether adaptations of RS would provide incremental benefits compared to its original form. Meta‐analyses revealed that while culturally adapted Cognitive Behavioral Therapy (CBT) was found to be effective for REM groups, including AANHPI individuals, in alleviating various mental health symptoms such as depression (e.g., Huey et al. 2023), only a handful of studies (e.g., W. C. Hwang et al. 2015) found the culturally adapted versions to be *more* effective than standard CBT treatments. In fact, a large number of studies testing the effectiveness of culturally adapted CBT were done in the absence of a point of comparison (Huey et al. 2023). In other words, there is sparse and tentative evidence supporting the incremental validity of cultural adaptations for some EBTs. The dissemination of adaptations should be informed by evidence showing lower effectiveness with a targeted population. Adaptations unsubstantiated by their incremental validity may jeopardize the fidelity of RS and reduce its effectiveness.

#### 3.1.3 Culturally Adapted Interventions Versus Culturally Responsive Interveners

Importantly, interventions are not delivered in a vacuum. The interveners themselves are an important part of the therapy outcome. Thus, studies are needed to determine whether formal intervention adaptation or cultural responsiveness training to individual clinicians may be more effective in addressing cross‐cultural differences. In the case of RS, it remains unclear whether formal adaptations should be applied to the treatment manual to match the needs of AANHPI families. Formal cultural adaptations may pose a risk of “othering” REM groups, amplifying the boundaries between groups with an “in‐group” versus “out‐group” perspective (Lekas et al. 2020). As an alternative approach, Huey et al. (2023) noted the importance of training clinicians to be more culturally responsive at a personalized level, rather than making a general adaptation to treatment manuals to be implemented for diverse individuals. This is analogous to the rationale of developing empirically supported clinicians rather than empirically supported treatments. Similar to how psychological treatment is not a “one‐size‐fits‐all” process, adaptations to interventions may be more effective if made (1) based on the unique circumstances of individual clients and sessions, and (2) collaboratively, with the understanding that clients are the experts of their own lived experiences. Training to increase individual practitioners' cultural humility, such that curiosity and openness to learn about others (i.e., other orientations to their clients) and self‐reflection and awareness (i.e., of clinicians' own assumptions and biases) may create a more personalized and effective approach.

### 3.2 Potential Reasons for Adaptations

In contrast, adapting RS for use with AANHPI clients may be helpful because of differences in the perception of savoring approaches and positive emotions that could result in lower engagement or effectiveness among AANHPI clients. Most savoring interventions were developed from a Western perspective and tested in White populations, raising questions about applicability to Eastern cultures (Smith et al. 2019). Indeed, the effectiveness of specific positive psychology practices differs across cultures: practices such as expressing gratitude were found to be less effective for AANHPI clients compared to White clients, while effects are equivalent for performing acts of kindness (Ng and Ong 2022). Given these findings, we propose the following considerations that indicate a potential need for adaptations.

#### 3.2.1 Cultural Differences In Perceptions of Savoring Approaches

RS adopts the savoring approach of *sharing with others* (Bryant and Veroff 2007), a strategy that involves sharing and celebrating a positive experience with others, which in turn amplifies the positive emotions associated with the memory (Gable et al. 2004). In RS, clients share the chosen memory with the intervener. This savoring strategy may be less effective for some AANHPI individuals (Smith et al. 2019), as previous studies found that Korean adults were less likely to share positive events due to concerns about jealousy damaging social relationships (Choi et al. 2019), potentially limiting the extent to which they might capitalize on sharing positive experiences with others. Adaptations of RS can address this cultural difference in orientation toward sharing positive experiences with others. For example, the intervener could frame the rationale for sharing positive attachment experiences as strengthening social connectedness rather than celebrating individual success, thus minimizing concerns for jealousy. RS could also complement the sharing with others savoring approach with counting blessings (Emmons and McCullough 2003), a savoring approach designed to cultivate gratitude toward positive aspects, by fostering clients' *contentment* related to the savored memory.

#### 3.2.2 Cultural Differences in the Perception of Positive Emotions

The current RS protocol formally addresses the coexistence of positive and negative emotions related to savored memories. The intervener elicits memories available to savor for the intervention by asking clients to recall memories that contain a strong positive emotion focus (memory selection), and then to focus on one of these memories in the memory reflection phase. To have memories that are suitable for savoring in RS, one intentionally notices the details of one's positive experience and constructs a lasting memory associated with the experience, which Bryant and Veroff (2007) refer to as *memory building*. Memories suitable for savoring in RS often contain negative emotions (e.g., anxiety, fear) because poignant moments in attachment relationships can be preceded by negative emotions.

Empirical evidence is needed to suggest whether the current approach to addressing emotion is sufficient for AANHPI individuals, who perceive positive emotions differently from White individuals. White individuals tend to perceive positive emotions as predominantly positive, while East Asian individuals perceive them more dialectically, acknowledging the potential coexistence of negative aspects of their experiences (Uchida and Kitayama 2009). For example, a Chinese saying from *Tao Te Ching* states: “福兮祸所伏, 祸兮福所倚,” which means positives hide within negatives, and negatives lean on positives (Laozi [ca. 400 BCE, Tao Te Ching]). Recognizing this interconnectedness, East Asian individuals appear more likely to down‐regulate positive emotions (Miyamoto and Ma 2011). Future research should examine whether RS's current approach to emotions aligns with the cultural sensibilities of AANHPI individuals, or whether more can be done to develop culturally attuned practices.

If evidence suggests a need for adaptation, the construction of a dialectical narrative of positive memories, acknowledging and accepting the coexistence of negative emotions, may be more congruent with AANHPI individuals' perception of emotions. RS could be adapted to more explicitly allow for memories that contain a mingling of emotional experience. Second, in RS, interveners often assist clients to be able to acknowledge the negative emotion and be able to set it aside to enable deeper processing of the positive emotion (Borelli 2024). By putting positive emotion at its central focus, interveners could potentially incorporate cultural sayings and metaphors describing the interplay of positive and negative emotions to bring awareness to their coexistence, while facilitating the *acceptance* of negative emotions. Finally, potential adaptations could have interveners prepared to address negative emotions, such as guilt, co‐occurring with positive emotions.

#### 3.2.3 Cultural Differences in Emotion Expression

Another consideration behind potential RS adaptations lies in the cultural differences in emotion expression. Compared to White Americans, AA showed lower emotion expressivity (verbal or non‐verbal expressions of perceived emotions) and more frequent emotion modification (intentional expressions of other emotions with or instead of the perceived emotions; H. S. Hwang and Matsumoto 2012). These differences can be attributed to cultural influences such as display rules (Ekman and Friesen 1969), family influences such as emotion socialization (Saw and Okazaki 2010), or individual differences such as motivation to maintain the relationship (H. S. Hwang and Matsumoto 2012). Several adaptations may be suitable to address these differences. More time could be allotted at the beginning for rapport building to properly set the stage for discussing emotions and relationships. The manual could potentially include a section on cultural differences in emotion expressions (e.g., Hmong Americans showed fewer non‐Duchenne smiles when expressing happiness; Tsai et al. 2002), such that interveners could adjust expectations on the type of emotion expressions to look for.

#### 3.2.4 Consideration of Acculturation Within AANHPI Families

Additionally, acculturation may occur at different rates for different members of a family; a mismatch in behaviors, values, and identities can result between generations (Ho 2014), suggesting a need to adapt RS to consider acculturation mismatches within families. Acculturation gaps can include differences in language proficiency, adherence to traditional cultural beliefs, and sense of belonging. Acculturation gaps predict elevated intergenerational cultural conflicts (Portes and Rumbaut 2006), which in turn predict poor psychological outcomes across the family system (Bamaca‐Colbert et al. 2019). For AANHPI caregivers, mismatches between traditional caregiving values and greater acculturation can even shape perceptions of caregiving and caregiving burden (Guo et al. 2019). Possible drivers of these differences may be related to personal characteristics, such as motivations (individualistic vs*.* collectivistic) and perception of positive emotions (solely positive *vs.* dialectical; Smith et al. 2019), as well as systemic aspects, such as intergenerational differences in values, behaviors, and acculturation.

#### 3.2.5 Potential Differences in Active Ingredients Across Cultures

Another potential reason to adapt RS is that while RS has been found to be effective in diverse samples with representations of AANHPI participants (e.g., Pereira et al. 2021), the active ingredients of RS may differ across cultures. For example, some may find the “relational” aspect of RS more salient through centering on a moment of interconnectedness, while others may find the “savoring” aspect more salient through attending, appreciating, and augmenting a positive experience. Similarly, some may find the initial process of recalling many memories to be more meaningful, while others may find the later process of diving into one memory more impactful. Some may value the process of focusing on the past experience, while others may value the part to look into the future. An important part of developing a form of RS that is attentive to the needs of AANHPI caregivers involves understanding what works for AANHPI specifically and not assuming that it would be similar to the overall population.

Taken together, there are a number of features of RS that may be culturally resonant with AANHPI clients, as well as other features that may land less favorably. Thus, it may be fruitful to explore and test cultural adaptations to RS with this unstudied sample. Next, we propose a research framework grounded in participatory research, targeting acceptability, accessibility, and effectiveness of RS in improving target outcomes among AANHPI communities.

## 4 Culturally Adapting RS Using a Participatory Research Approach

While intervention responsiveness to individuals' contexts and identities appears to carry important potential benefits (e.g., acceptability, effectiveness, engagement), the decision whether or not to make adaptations for use with specific populations is a sensitive one and must be done with care. As we have discussed, inaccurate assumptions about the needs of underserved communities that dictate the types of care they do or do not receive can result in immediate and long‐lasting harm. In this section, we discuss the steps that can be taken to inform the process of determining if cultural adaptations are indicated and, if so, how they should be undertaken.

For researchers who do not belong to the target population or work with a particularly under‐researched group, qualitative methods such as interviews and focus groups may position researchers to listen more carefully to the narratives of their participants. Specifically, researchers may adopt a CBPR framework (Parra‐Cardona et al. 2023) to facilitate decision‐making around adaptation and to ensure that communities are represented in the research process. A CBPR approach can assist in the possible adaptation and delivery of RS to AANHPI samples, which starts from the study design and extends across all stages of research. We outline several high‐level steps toward the ultimate goal of delivering RS to AANHPI caregivers in an accessible and acceptable manner, building on the Formative Method for Adapting Psychotherapy (FMAP; W. C. Hwang 2009), which consists of five phases: (1) community‐based generation of knowledge with relevant stakeholders; (2) incorporate bottom‐up knowledge with top‐down knowledge; (3) iterative feedback and revision process with stakeholders; (4) testing adaptation; (5) finalizing adaptation.

### 4.1 Phase 1: Generate Knowledge with Community Stakeholders

At the heart of CBPR are partnerships, wherein researchers work to intentionally build trust with community organizations. In research contexts, community relationships are often relegated to the background as a recruitment mechanism that facilitates data collection and participant retention. However, the translation, dissemination, and implementation of research findings may also profoundly benefit from the inclusion of community voices. A carefully developed community partnership is particularly important in carrying out research with underserved groups, many of which have fraught or distrusting relationships with educational or research institutions (Di Bari et al. 2007). Community partnerships may improve access to what researchers often deem “hard to reach” groups and reduce the potential for further harm to be inflicted upon already vulnerable communities. AANHPI communities in particular have been found to be particularly hesitant compared to other racial‐ethnic groups to be involved in research and also to report lower rates of fair compensation (Liu et al. 2019). In selecting a community partner, researchers may seek out organizations that are accessible, non‐stigmatizing, and have invested resources and time into building long‐standing relationships with the local community. However, not all community‐serving organizations play the same or similar roles despite sharing some of these previously outlined features and should be assessed for appropriateness. Possible sites of interest for the delivery of RS to AANHPI caregivers may include churches, temples, libraries, community health centers, schools, afterschool centers, and other community‐serving organizations. Relationship building, therefore, may look different across groups depending on culturally salient sites or individuals. For example, churches serve as cultural and social hubs for many Korean Americans, and Native Hawaiians may be approached via native cultural brokers. Still, it is important to emphasize that not all members of an ethnic group may respond to the same relationship‐building or recruitment strategies.

Other community‐based intervention development studies have been successful by incorporating community members and the target population into the development and delivery of the study. Baker et al. (2018), for example, developed a violence prevention‐based intervention for young Black emerging adult men, delivered by barbers in barbershop settings; their CAB was composed of barbers and barbershop owners, who were crucial to the delivery and implementation of the intervention. Given the focus of the present paper, researchers should recruit individuals representative of the target sample (e.g., AANHPI caregivers) and/or relevant stakeholders (e.g., community health or service workers embedded in predominantly AANHPI neighborhoods or cultural/ethnic enclaves) to form a (paid) community advisory board (CAB). Given the novelty of this intervention with AANHPI populations as well as the heterogeneity of AANHPI groups, it may be necessary to begin efforts towards introducing and examining the use of RS with a single ethnic group, and perhaps even further specified by acculturative status or other shared characteristics. Specifying exactly the group of interest can increase the likelihood of gathering a relatively representative CAB. The community partner may provide further insight into possible outcomes to target, particularly under‐resourced members of the community, and what past community or research efforts have and have not been fruitful. Doing so can allow researchers to maximize the impact of their study and streamline the allocation of study resources, all the while minimizing the potential harm of their engagement with the community.

### 4.2 Phase 2: Incorporate Bottom‐Up and Top‐Down Knowledge

Before adapting RS, it is necessary to first assess the acceptability and efficacy of standard RS among AANHPI caregivers. Examining the effectiveness of the original intervention avoids the common assumption that REM groups warrant unsubstantiated modifications or adaptations based on cultural differences. The CAB would guide the recruitment considerations at this stage (e.g., where to recruit, language used, framing, and feedback on recruitment materials). A simple early adaptation—translation of recruitment and intervention manuals—may already be necessary depending on the target population and language needs. Interveners, ideally embedded in the community themselves, can administer one round of RS to participants, either in‐person at an accessible setting or virtually, and then gather preliminary acceptability ratings and qualitative feedback via individual interviews. Two findings may serve as further support for adaptation: (1) the impact of RS on well‐being, relationship, and satisfaction outcomes for AANHPI participants is less than that of other racial‐ethnic groups and/or (2) qualitative feedback points to unique barriers or participant qualms with RS content.

Following a full course of RS, researchers should compare the efficacy of the intervention in shifting dimensions of interest that have been previously tested in other groups and compare effect sizes to determine if RS works as well for AANHPI participants as they have for previous samples. To examine barriers and facilitators to delivering RS, researchers may also consider forming a focus group with interveners. After presenting the intervention materials to the group, feedback should be elicited regarding possible barriers and facilitators for treatment delivery, engagement, and implementation. Researchers should expect to make continued modifications and maintain flexibility at this stage. At this stage, researchers should thoughtfully reflect on the tangible resources that the community can retain after the study concludes (e.g., a training manual, recorded workshop, etc.).

### 4.3 Phase 3: Iterative Feedback and Revision Process with Stakeholders

If researchers find that adaptations are needed, the next objective should be to synthesize participant feedback and present findings to the CAB to identify possible targets for adaptation and the degree to which the RS intervention may be adapted. More specifically, adaptations may address surface structures and/or deep structures of an intervention (Resnicow et al. 1999). Resnicow et al. (1999) emphasize that surface structure contributes to the acceptance of messages or feasibility, while deep structure contributes to salience or broader intervention impact. By this stage, it is likely that at least one surface‐level feature—language—may have already been implemented. Other surface‐level features may include intervention content such as cultural imagery (i.e., drawn from foods, traditions, idioms) that may contribute to intervention acceptability based on recognizable elements and overall “fit” with the target culture. Many intervention adaptations stop here without further delving into deep‐level features, which expand to consider intersecting systems that act upon and contribute to health behaviors (e.g., cultural values, norms, perceptions). While it is possible that superficial adaptations around cultural fit may be sufficient in some cases, it is also important to consider the deep structure features of the intervention, such as its manner of delivery and implementation, to maximize its feasibility, fidelity, and longevity within the target population.

An important balance should be struck to ensure that RS is responsive to cultures as well as individual clients while maintaining its core. The aforementioned approaches seek to enhance cultural responsiveness. At the same time, we emphasize three core RS components that should be maintained across adaptations: (1) memory selection, (2) memory reflection following five steps, and (3) the focus on positive memory with strong attachment content. Adaptations to the RS manual can be enhanced by intentionally selecting interveners who are well‐matched to the target population and permitting flexible delivery rather than strict adherence to the script. This approach leverages the language and cultural knowledge of individuals with deep ties to the community, enabling more personalized and effective implementation.

### 4.4 Phases 4 and 5: Testing and Finalizing Adaptation

Upon finalizing the adaptations to RS, researchers can take the oft‐missed step of comparing the standard or original intervention to the culturally adapted version. At this stage, it is essential not to hold on too tightly to the assumption that the culturally adapted version is necessary or will be better for the particular sample, let alone AANHPI individuals more broadly. To our knowledge, no randomized controlled trials (RCTs) have been conducted comparing culturally adapted RS to the original version of RS. However, a major challenge is honing in on effects for specific Asian ethnic subgroups. For instance, what is efficacious for Chinese Americans may not be efficacious for Native Hawaiians. That is, an adaptation for AANHPI may not meet the specific ethnic subgroup needs. Researchers may consider conducting a single or multi‐arm trial comparing standard RS, culturally adapted RS, and/or a control group. Upon completion of the study, researchers may again work with the CAB to determine the best avenues and formats for the dissemination of research findings (e.g., community fair, newspaper article, blog post, animated video on social media, digital resource) and collaborate with significant CAB or community partners in writing scientific or policy‐focused articles.

## 5 Conclusion

RS has significant potential to augment the strengths of AANHPI caregivers and address their pressing clinical needs, with its connections to systems theories and its relational, strengths‐based focus. As the field of SFT moves toward enhanced cultural responsiveness, we offer these considerations as a means of moving the field forward. While our comments focus on RS, many of the considerations suggested here could be applied to other systems‐focused interventions that show promise for adaptation or integration for this population. Future work with RS may expand its potential for effecting third‐order change by considering broader systems and ecological contexts beyond family or immediate communities.

## Endnotes

1 The Asian American population in the United States consists of great diversity in terms of country of origin. Thus, in the current paper, we use the term “Asian American, Native Hawaiian, or Pacific Islander (AANHPI)” to capture the broad diversity of the community.
